# A call to develop rigorous standards for excellence in idiopathic intracranial hypertension: RISE-IIH

**DOI:** 10.1038/s41433-026-04358-8

**Published:** 2026-04-07

**Authors:** Daisy MacKeith, Bethany Ellis, Jessica Katanga, Arani Nitkunan, Alis Sejourne, Sonia MacDiarmid, Alysha Budd, Claire Voas-Clarke, Gabriele Berman, Susan P. Mollan, Joanne Adeoye

**Affiliations:** 1 Department of Orthoptics, Cambridge University Hospitals NHS Foundation Trust, Cambridge, UK; 2 Glenfield Hospital, Leicester Partnership NHS Trust, Leicester, UK; 3 Neurology department, Croydon Health Services NHS Trust, Croydon, UK; 4 Department of Orthoptics, The Royal London Hospital, London, UK; 5 Department of Orthoptics, Ophthalmology, Warrington and Halton Hospitals NHS Foundation Trust, Warrington, UK; 6 Department of Orthoptics, Countess of Chester Hospital, Chester, UK; 7 Birmingham Neuro-Ophthalmology, University Hospitals Birmingham NHS Foundation Trust, Birmingham, UK; 8 Ophthalmology Department, Queen’s University, Kingston, ON Canada; 9 Metabolism and Systems Science, School of Medical Sciences, University of Birmingham, Birmingham, UK; 10 Department of Orthoptics, School of Allied Health Professions and Nursing, University of Liverpool, Liverpool, UK

**Keywords:** Health services, Diagnosis

Idiopathic intracranial hypertension (IIH) is an increasingly prevalent condition characterised by raised intracranial pressure, papilloedema and headache with emerging evidence suggesting a systemic metabolic disorder [1, 2]. People with IIH have cited challenges in their care and made recommendations in key areas such as communication regarding the relationship between weight management, disease development and remission [3]. A person with IIH may meet many specialists and therefore a coordinated standard approach to care is recommended [4]. The first consensus guidelines for IIH were published in 2018 but how these have translated into clinical practice remains largely unknown [4, 5]. To understand what quality metrics are important to health care professionals, herein we summarised eight different audits that have been performed, all of which included standards selected from the IIH consensus guidelines [4].

Up to 60% of neuro-ophthalmology services within the United Kingdom (UK) have developed clinical pathways for IIH, with allied health professionals running dedicated clinics. Therefore, following a literature review, a scoping call via the British and Irish Orthoptic Society (BIOS) neuro-orthoptic clinical advisory group mailing list, was performed. Data shared from National Health Service (NHS) Trust approved audit results in addition to published audits has been collated. The audits were from different settings, including an equal split of superregional, regional teaching, and local general hospitals; and include ophthalmology, eye casualty and neurology departments. All audits were conducted after the publication of the IIH consensus guidelines [4], with three including some data from cases identified prior to 2018. For the purpose of this analysis, the individual NHS Trusts are not identified. The audits included 469 people diagnosed with IIH. Thirty-three standards were included across the audits.

To aide visualisation, summary data from all eight audits is tabulated (Figure 1) and the data were graded with traffic light colours featuring completeness: dark green represented 100% compliance; light green indicated 99-75% compliance; yellow represented between 74-50% compliance, which was below target; and those in red were considered to be significantly below target compliance at 49% or less. Only one Trust, auditing an orthoptic-led clinic, achieved above 75% compliance with all but one of its reported standards. There was no one standard universal to all eight audits.

**Fig. 1:**
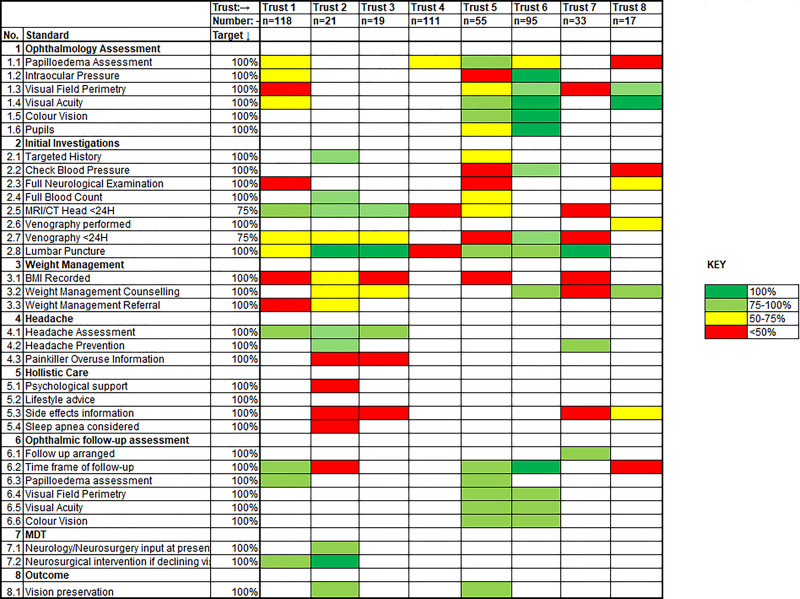
Tabulated Data from Audits of IIH Care Across 8 UK NHS Trusts against the 2018 IIH Consensus Guidelines. The table shows varying recording and compliance with the 33 audit standards across the 8 NHS Trusts. Please see Appendix 1 for further detail of each standard. The majority of points measured are shown as above 75% (green), however, only 7 standards met 100% compliance (dark green) and a significant proportion of standards are shown as significantly below target <49% (red).

All audits captured data on initial investigations. Lumbar puncture is crucial for securing a definite diagnosis [4–7]. However, only five centres met target compliance. There was good compliance in obtaining neuro-imaging within 24 hours but poor compliance with obtaining venography. Venography remains an essential investigation to rule out cerebral venous sinus thrombosis [5, 8]. There was poor compliance in documenting cranial nerve examination in the three audits that sampled this.

Quality of the ophthalmology assessment was the focus of four audits, and three centres fell below or significantly below standard for recording visual field assessment at presentation. This is of particular concern as formal visual fields are recommended to help guide management decisions [8]. Follow-up ophthalmic data were more complete but did not meet target in two of five audits.

Weight management is pivotal to disease control, and it has been found that self-reported body mass index (BMI) is underestimated by people with IIH and controls [3, 9–11]. No audit adequately recorded a height and weight measurement at presentation, despite raised BMI being the main risk factor for developing IIH [12, 13]. This is concerning as it shows that little has changed despite the publication of the IIH guidelines [4]. Education for health care professionals regarding communication of body weight and its management has been identified by IIH UK, a patient charity that supports carers and patients living with IIH as an unmet need [3]. Compliance with weight-management counselling was above target in only two audits, one of which was the orthoptic-led clinic, likely due to a formal protocol being established. Unfortunately, there was poor compliance with formal onward referrals to weight management services and this should be highlighted as a significant barrier for patients [14]. The National Institute of Health and Clinical Excellence (NICE) has recently recognised IIH as a co-morbid condition in relation to tier 3 weight management services and a condition which can improve after bariatric surgery [15].

Despite being the most common symptom of IIH and the least successfully treated [16, 17], evaluation of headache was only performed in three audits. Patient education relating to medication overuse headache was either not included as a standard or, when captured, was significantly below target. Holistic care standards that included counselling women about teratogenic side-effects of medication were not included in four of the audits and significantly below target in three.

Overall, we found that there was a wide variation in which standards are being audited [17]. Poor adherence to standards in key diagnostic tests highlights an urgent need for reform of care pathways for people with suspected IIH to reduce diagnostic error, a concern that has previously been highlighted [18, 19]. This analysis suggests that significant improvements could be made in hospital services to enhance the equity and quality of care for people with IIH in the UK.

To impact care for people with IIH, it is important to involve stakeholders that have the knowledge and ability to implement best practice. A coordinated approach is required to deliver equitable care across the UK. With the emphasis on patient-centred care in the NHS 10-year plan [20] and supported expansion of roles under the advancing practice agenda, non-medical professionals now fill key roles in IIH care, often co-ordinating joined-up care and communication between services [6, 21]. The results of this analysis underline the potential benefits of introducing national quality standards for IIH, as exists for other conditions [22–25]. We propose a call to develop Rigorous Standards for Excellence in Idiopathic Intracranial Hypertension: RISE-IIH. These quality standards should be developed by multidisciplinary health care professionals, with involvement of people living with IIH. The impact would be to influence commissioning bodies, provide auditable metrics to bench-mark services against and aim to build equitable care across the UK, reduce known barriers, identify blind spots and improve patient outcomes.

## Supplementary information

Supplementary Appendix
